# Kommerell Aneurysm

**DOI:** 10.5334/jbsr.2375

**Published:** 2021-01-04

**Authors:** Maïté Godfrin, Nigel Howarth, Denis Tack

**Affiliations:** 1 EpiCURA, BE; 2 Clinique des Grangettes, CH

**Keywords:** Kommerell aneurysm, Kommerell diverticulum, mediastinal enlargment, Chest X-ray, Chest-CT

## Abstract

**Teaching Point**: Kommerell aneurysm is a rare differential diagnosis of mediastinal enlargement on a chest radiograph that requires CT for accurate diagnosis.

## CASE HISTORY

**Figure 1:**
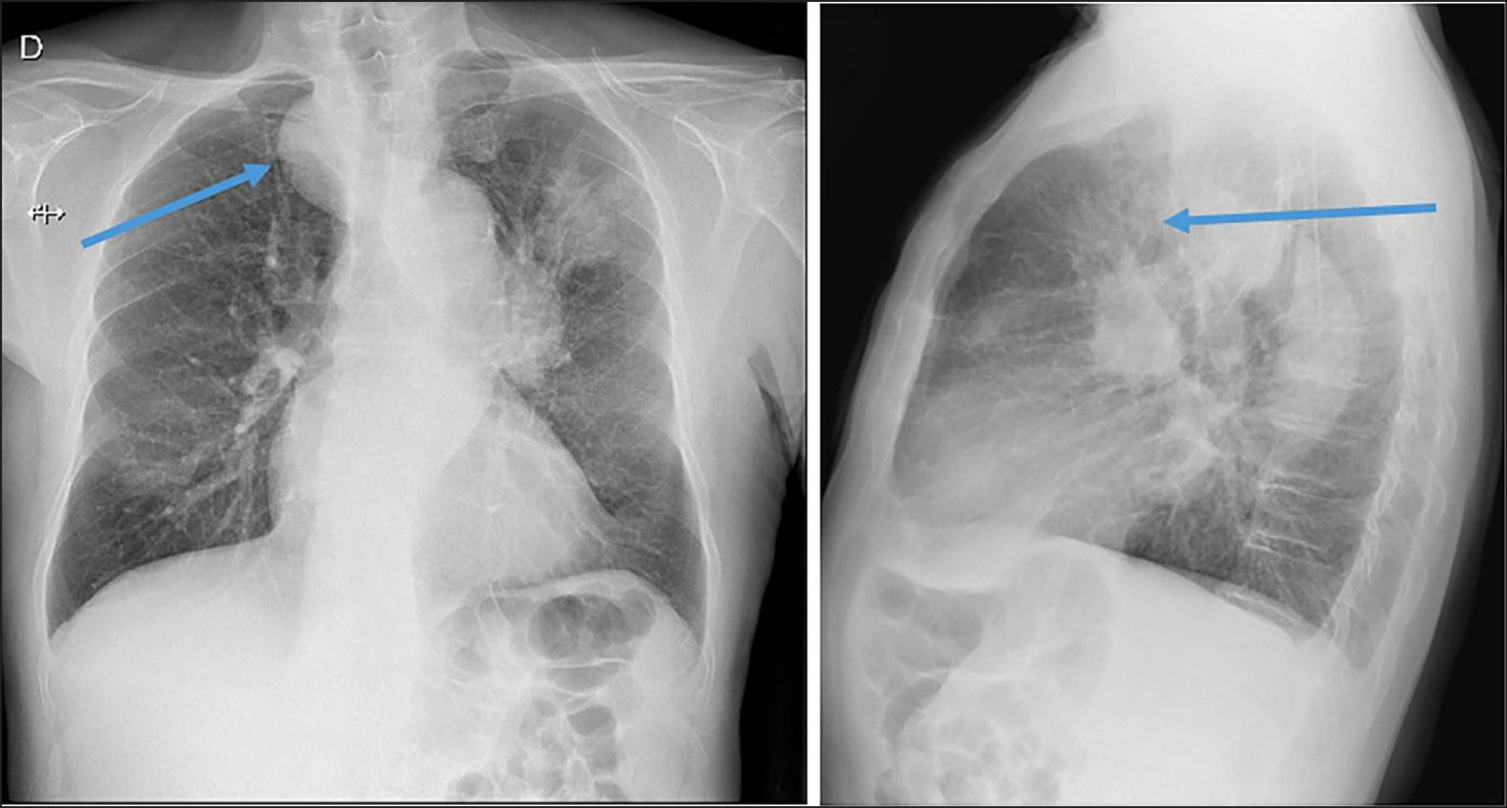


A frontal and lateral chest radiograph was obtained in a 73-year-old man with hemoptysis (Figure 1). It revealed a 4 cm nodular lesion in the left apex, a left hilar mass in the aortopulmonary window, an enlargement of the right paratracheal stripe on the frontal view, and an anterior displacement of the trachea on the lateral view (arrows). While the two first lesions most likely represented lung carcinoma with hilar lymphadenopathy, the right paratracheal abnormality was first thought to be due to more lymphadenopathy or a goitre.

A chest computed tomography (CT) was performed the same day, confirming a pulmonary mass in the left upper lobe with lymphadenopathy in the aortopulmonary window. The right paratracheal lesion, however, proved to be an unsuspected aneurysm developed on an arteria lusoria (anatomical variant of right subclavian artery) named “Kommerell aneurysm.”

**Figure 2:**
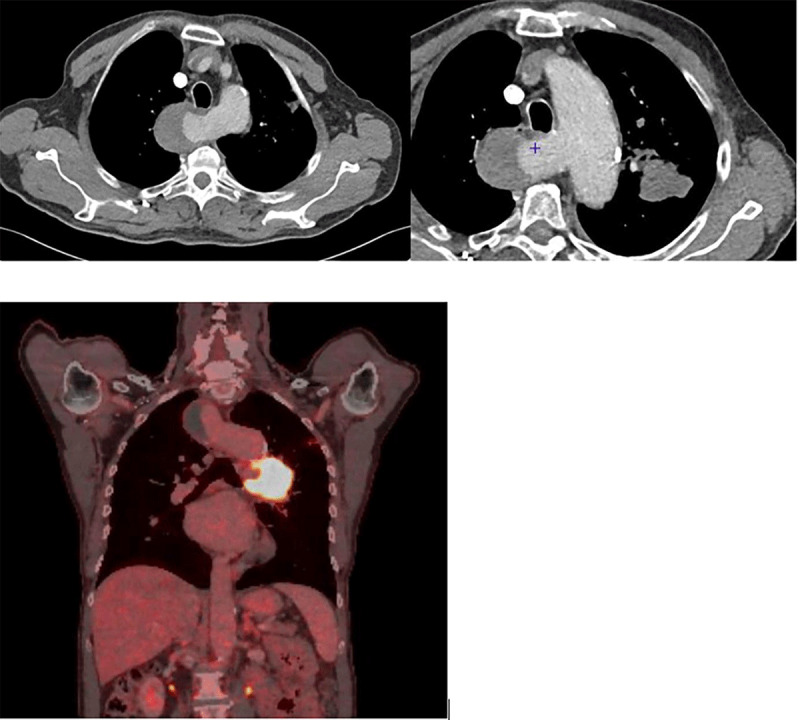


The positron emission tomography (PET) CT (Figure 2) was positive in the aorto-pulmonary window. Combined chemotherapy and radiation therapy was proposed. The Kommerell aneurysm was not operated.

## COMMENTARY

Aberrant right subclavian artery is an anatomical variant, affecting 1% of the population. Kommerell diverticulum is present in about 60% of cases and is described as a diverticulum at the origin of an aberrant right or left subclavian artery in a left-sided or in a right-sided aortic arch, respectively [1].

Aneurysms of this diverticulum (Kommerell aneurysm) are seen in 3–8% of cases. These aneurysms present as a fusiform dilation of the diverticulum at the origin of the aberrant subclavian artery [1].

Most patients are asymptomatic but can present with dysphagia, dyspnea, or chest pain. These symptoms depend on the location and size of the aneurysm (retro-esophageal, between esophagus and trachea or pre-tracheal).

Kommerell aneurysm requires surgical treatment because of the risk of rupture and dissection. Rupture can occur in approximately 19% of cases [1].

Uncomplicated diverticulum of Kommerell, saccular aneurysm of the thoracic aorta and ductus diverticulum are mimics of Kommerell aneurysm. The differential diagnosis is based on the origin of the aneurysm, outpoutching contents, and morphology. The Kommerell diverticulum arises from the aortic arch and the Kommerell aneurysm from the Kommerell diverticulum. Saccular aneurysms of the aortic arch do not involve the origin of the aberrant right subclavian artery. Ductus diverticulum is located at the level of the ligamentum arteriosum (inferior anteromedial aspect of the aorta). The Kommerell aneurysm may be saccular or fusiform. The Kommerell diverticulum is always fusiform.
